# ApoE Isoform-Dependent Effects on Extinction of Contextual Fear Memory and Passive Avoidance Memory

**DOI:** 10.3390/ijms26125820

**Published:** 2025-06-17

**Authors:** Elizabeth Saltonstall, Alexandra Pederson, Abigail O’Niel, Sarah Holden, Kat Kessler, Eileen Ruth Samson Torres, Jacob Raber

**Affiliations:** 1 Department of Behavioral Neuroscience, Oregon Health & Science University, Portland, OR 97239, USA; saltonse@ohsu.edu (E.S.); pedersoa@ohsu.edu (A.P.); oniela@ohsu.edu (A.O.); holdens@ohsu.edu (S.H.); katkessler9@gmail.com (K.K.); est4003@med.cornell.edu (E.R.S.T.); 2 Departments of Neurology and Radiation Medicine, Division of Neuroscience, ONPRC, Oregon Health & Science University, Portland, OR 97239, USA

**Keywords:** depressive-like behavior, tau, genetic risk factors, avoidance, PTSD

## Abstract

Following exposure to trauma, avoidance behavior can be protective but also contribute to severe symptoms and interfere with exposure-based therapy. Extinction of fear conditioning by exposure to the same environment or environmental cues that were present during the initial traumatic event but without including the aversive stimulus or stimuli is often used to study post-traumatic stress disorder (PTSD), a condition characterized by an inability to suppress conditioned fear responses. A limitation of this paradigm is that one cannot avoid the context or cues associated with the initial traumatic event. In contrast, in the passive avoidance test, one can escape the environment associated with the aversive stimulus. Genetic factors might modulate the ability to extinguish fear memory. In this study, we compared the effects of distinct human apoE isoforms on the extinction of contextual fear and passive avoidance memory, as well as on subsequent activity levels, depressive-like behavior, and hippocampal levels of tau, in targeted replacement mice.

## 1. Introduction

Following exposure or re-exposure to one or more traumatic events, some of those exposed develop post-traumatic stress disorder (PTSD), a disorder with symptoms including intrusive recollection of the traumatic events and avoidance of trauma-related cues [1,2]. When re-exposed to the same environmental cues that were present during the traumatic event, those affected show an impaired extinction of fear and often an avoidance of trauma-related cues [3,4]. While avoidance behavior is protective, it can contribute to severe symptoms and was proposed to interfere with exposure-based therapy [4,5]. However, protective avoidance has positive effects that can also be considered active coping [6,7] and serve an adaptive function that might even be beneficial if included as part of exposure therapy [8,9,10,11]. Therefore, more research is needed to increase our understanding of the factors that influence coping mechanisms.

In humans and pre-clinical animal models, extinction of fear conditioning by exposing the humans or animals to the same environment or environmental cues that were present during the initial traumatic event but without including the aversive stimulus or stimuli is often used to study PTSD [12,13,14,15,16,17,18]. An inability to suppress conditioned fear responses is the hallmark of PTSD [19,20]. Pre-existing factors modulate the ability to extinguish conditioned fear. For example, a smaller hippocampal volume is a risk factor to develop PTSD, and hippocampal volume is also negatively associated with disease severity [21]. A larger volume of the left amygdala and, within the hippocampus, a smaller volume of the subiculum (left and right) and the right CA1 region might especially be a risk factor to develop non-remitting PTSD [22]. The thalamic nucleus reuniens–hippocampal CA1 region pathway is important for the disruption of the retrieval of contextual fear memories in the hippocampal CA1 region and the extinction of contextual fear memories [23].

Genetic factors also might predispose an individual to develop PTSD and/or more severe PTSD. Apolipoprotein E (apoE), which is involved in cholesterol metabolism and repair after injury, exists in humans as three major isoforms; E2, E3, and E4 [24]. Compared to E3, E4 increases the risk of developing Alzheimer’s disease, while E2 reduces the risk of developing Alzheimer’s disease [25]. Regarding PTSD, there is support for an increased risk of developing PTSD in E2 carriers [26,27,28] and E4 carriers [29,30,31]. Consistent with these human data, an impaired extinction of contextual fear was observed in young adult (4–5- or 3–6-month-old) singly housed human E2-targeted replacement mice expressing human apoE under control of the murine apoE promoter [26,32]. It is unclear whether there are apoE isoform-dependent effects on the extinction of contextual fear in older mice. This is important as PTSD is associated with accelerated age-related cognitive decline [33] and risk of developing Parkinson’s disease [34].

There is increasing support for the idea that rodents and farm animals like pigs demonstrate signs of empathy [23,35,36,37,38]. Empathy was moderated by genetic background in a cued fear conditioning task [39]. In C57BL/6J mice, empathic fear responses were only seen in mice that shared a similar experience (i.e., receiving a foot shock) 24 h earlier [40]. Consistent with the notion that empathy might enhance fear memories, freezing in a cued fear memory test [41] and in a contextual fear memory test [42] 24 h after fear learning was stronger in group- than single-housed C57BL/6J mice. However, single- and pair-housed Long Evans rats showed comparable freezing in contextual fear memory test 24 h after fear learning [43] and single- and group-housed C57BL/6J mice showed similar freezing in a discriminatory auditory fear conditioning task [44]. An open question is whether group housing modulates the apoE isoform-dependent effects on extinction of contextual fear memory.

While a lot has been learned about the extinction of conditioned fear using pre-clinical models of fear conditioning, a limitation of the fear conditioning paradigm is that the animal cannot avoid the context or cues associated with the initial traumatic event. This is important as those affected by traumatic events who developed PTSD often show avoidance of trauma-related cues [3,4]. This could be considered active coping [6,7] and serve an adaptive function [8,9,10,11]. In contrast to fear conditioning, in avoidance tests, animals can actively or passively escape the environment associated with the aversive stimulus. For example, in the passive avoidance test, the animal can choose to avoid the compartment in which it previously experienced an aversive stimulus by remaining in the other compartment. While, typically, memory retention in the passive avoidance test is only assessed at one time point following fear learning, Chen et al. re-exposed mice in the passive avoidance memory test over subsequent days [45]. An open question is whether apoE also has isoform-dependent effects on the extinction of passive avoidance memories or only on the extinction of contextual fear memories, a paradigm that lacks the ability to avoid cues associated with the initial traumatic event.

To examine the potential molecular synaptic markers associated with traits of PTSD, MAP-2, synaptophysin, and tau levels were assessed in the hippocampus. Microtubule-associated protein 2 (MAP-2), a dendritic protein important for stabilizing microtubuli and dendritic plasticity, is required for dendrite elongation [46]. MAP-2 levels show age-related changes in rodents [47,48] and nonhuman primates [49], are affected in mice who receive irradiation (X-rays, whole body, 4 Gy) 1 day following training for contextual fear conditioning and were tested for extinction over 8 days starting 14 days after training [50], and are increased in the hippocampus, cortex, and amygdala three months following ^137^Cs irradiation (head only, 10 Gy) [51]. Levels of synaptophysin, a synaptic vesicle-associated protein involved in the formation of synapses in cultured hippocampal neurons, correlate with cognitive performance and are altered in aged mice as well [47]; they also correlate with measures of anxiety and cognition in aged non-human primates [52]. Tau is a microtubule protein, important for stabilizing microtubule and synaptic physiology, including long-term depression [53]. Reducing hippocampal tau levels in adult mice impairs spatial memory retention in the water maze and motor coordination on the rotarod and is associated with reduced levels of synaptic proteins associated with learning and memory, including synaptophysin [54]. Hippocampal tau might be involved in the regulation of stress as well. Following social defeat stress, hippocampal tau levels are increased, which in turn is associated with increased anxiety [55]. It is unclear whether there might be differences in the levels of MAP-2, synaptophysin, or tau levels in the hippocampus, a brain area involved in both passive avoidance [56] and contextual [57,58] fear learning and memory.

In this study, we assessed (1) whether housing condition modulates the apoE isoform-dependent effects on extinction of contextual fear memory in young mice; (2) whether there are apoE isoform-dependent effects on the extinction of contextual fear in older mice; and (3) whether there are apoE isoform-dependent or age-dependent effects on the extinction of passive avoidance memory. Finally, we assessed whether there are apoE isoform- or test history (i.e., contextual fear or passive avoidance memory extinction)-dependent effects on measures of anxiety and/or depressive-like behavior or on hippocampal levels of synaptophysin, MAP-2, or tau.

## 2. Results

### 2.1. Study 1

**Figure 1:**
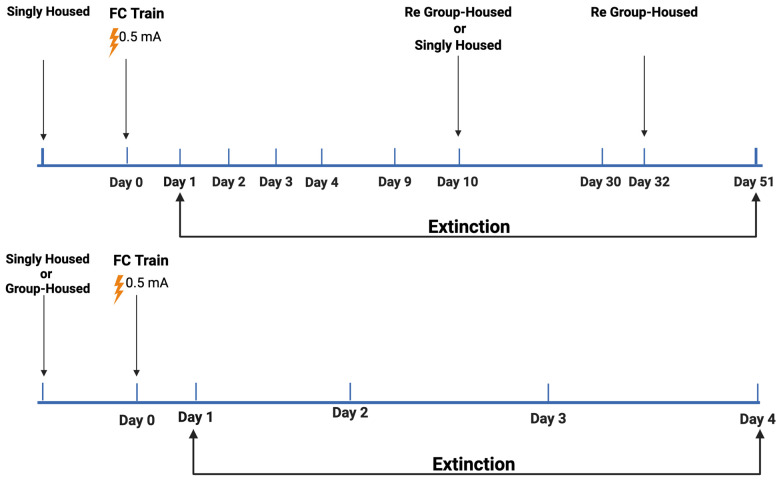
Experimental design of contextual fear extinction in young (**Top**) and middle-aged (**Bottom**) mice following training involving two shocks. For details, see main text. Created in Biorender. Bolis, M. (2025). Study 1 young: https://app.biorender.com/illustrations/65b40600847f4a7603a1ed0e?slideId=fdbe3221-ae37-4fd6-b8aa-758347bcf4ed (accessed on 11 June 2025); Study 1 middle-aged: https://app.biorender.com/illustrations/65aaed0e60000ca3be22d63a?slideId=fdbe3221-ae37-4fd6-b8aa-758347bcf4ed (accessed on 11 June 2025).

Study 1 (see Figure 1 for the experimental design) involved 234 mice (*n* = 117 mice/sex; *n* = 120 group-housed mice and *n* = 114 singly housed mice; *n* = 83 E2 mice, *n* = 85 E3 mice, and *n* = 66 E4 mice; *n* = 93 young mice and *n* = 141 older mice). The young mice (*n* = 93 mice, Figure 1 Top) consisted of *n* = 49 male and *n* = 44 female mice; *n* = 31 E2, *n* = 33 E3, and *n* = 29 E4 mice; *n* = 39 group-housed mice (2.0 ± 0.1 months of age) and *n* = 54 singly housed mice (3.2 ± 0.2 months of age). The group of young E2 and E4 mice was tested for an additional extinction day 9 days after the training day. The following day, half of the mice were group-housed and half of the mice remained singly housed and were re-tested 30 days following training. Two days later, the singly housed mice were group-housed as well, as they were housed originally, and tested for extinction 51 days following training. The goal of varying the housing condition and assessing extinction at late time points was to assess whether housing conditions affect the extinction of fear memories and whether the established memories are long-lasting.

The middle-aged mice (*n* = 141 mice, Figure 1 Bottom) consisted of *n* = 68 male and *n* = 73 female mice; *n* = 52 E2, *n* = 52 E3, and *n* = 37 E4 mice; *n* = 81 group-housed mice (8.7 ± 0.2 months of age) and *n* = 60 singly housed mice (11.7 ± 0.5 months of age). To determine whether the housing condition during training and the first four days of extinction affects the percent freezing, the mice were singly or group-housed prior to the training.

#### 2.1.1. Baseline Activity Levels During Fear Learning

When the activity levels during the baseline period (i.e., prior to the first shock during training) were analyzed, there was no effect of genotype, age, or housing but there was an effect of sex (*F*(1,210) = 7.272, *p* = 0.008), with higher activity levels in females than males (Figure S1).

#### 2.1.2. Response to Shocks During Fear Learning

When the response to the shocks (i.e., motion to the shocks) was analyzed, there was an effect of sex (*F*(1,210) = 33.514, *p* < 0.001), with a stronger response in males than females; age (*F*(1,210) = 33.913, *p* < 0.001), with a stronger response in young than middle-aged mice; a genotype × housing interaction (*F*(2,210) = 6.345, *p* = 0.002); a genotype × age interaction (*F*(2,210) = 4.586, *p* = 0.011); a shock × age interaction (*F*(1,210) = 13.499, *p* < 0.001); a shock × sex × age interaction (*F*(1,210) = 4.068, *p* = 0.045); and a shock × genotype x age interaction (*F*(2,210) = 5.202, *p* = 0.006) (Figure S2).

When the response to the shocks (i.e., motion to the shocks) was analyzed in the young mice only, there was an effect of shock (*F*(1,81) = 7.702, *p* = 0.007); an effect of sex (*F*(1,81) = 19.214, *p* < 0.001), with a stronger response to shocks in males than females; a shock × genotype interaction (*F*(2,81) = 3.235, *p* = 0.045), with a more differential response to the two shocks in E4 than E2 or E3 mice; and a genotype × housing interaction (*F*(2,81) = 3.260, *p* = 0.043) (Figure S2A–D), with a stronger response to the shocks in single- than group-housed E3 mice but a stronger response to the shocks in group- than single-housed E2 and E4 mice.

When the response to the shocks (i.e., motion to the shocks) was only analyzed in older mice, there was an effect of shock (*F*(1,129) = 7.267, *p* = 0.008); sex (*F*(1,129) = 13.707, *p* < 0.001), with a stronger response to shocks in males than females; genotype (*F*(2,129) = 4.787, *p* = 0.010), with a stronger response to the shocks in E4 than E3 mice; a shock × genotype interaction (*F*(2,129) = 3.826, *p* = 0.024), with a more differential response to the two shocks in E4 than E2 and E3 mice; a genotype × housing interaction (*F*(2,129) = 3.681, *p* = 0.028), with an increased response to the shocks in group-housed E2 and E4 mice than single-housed E2 and E4 mice; and a trend towards a shock × housing interaction (*F*(1,129) = 2.965, *p* = 0.087) (Figure S2E–H).

#### 2.1.3. Percent Freezing During the ISIs (Inter-Stimulus Intervals) During Fear Learning

When the percent freezing during the two ISIs, a measure of fear learning, was analyzed, there was an effect of ISI *F*(1,210) = 141.985, *p* < 0.001), with more freezing during the second than first ISI; an effect of housing (*F*(1,210) = 12.288, *p* < 0.001), with more freezing in single- than group-housed mice; a sex × genotype interaction *F*(2,210) = 5.487, *p* = 0.005), with more freezing in E2 female than male mice but less freezing in E3 and E4 female than male mice; and an ISI × genotype interaction *F*(2,210) = 3.697, *p* = 0.026), with a more pronounced difference in freezing during the two ISIs in E3 than E2 or E4 mice (Figure S3).

#### 2.1.4. Percent Freezing During the Extinction Days

**Figure 2:**
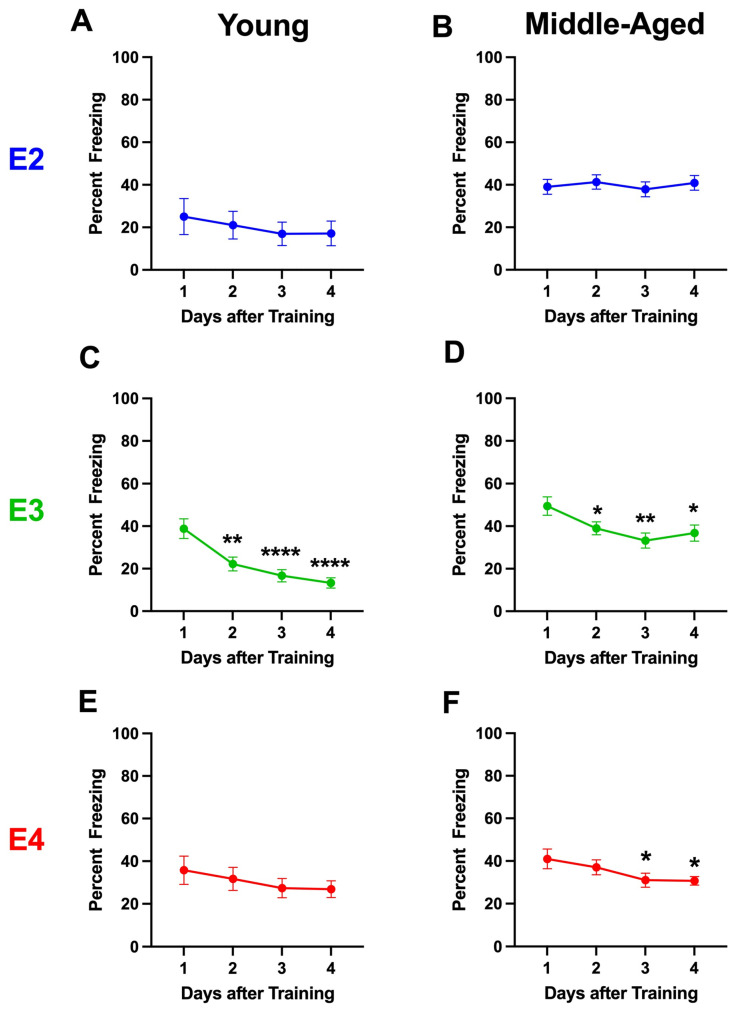
Extinction of contextual fear memory in young (**A**,**C**,**E**) and middle-aged (**B**,**D**,**F**) E2 (**A**,**B**), E3 (**C**,**D**), and E4 (**E**,**F**) group-housed mice. (**C**,**D**). When the extinction curves in young and middle-aged mice of each genotype were analyzed separately, there was only an effect of day in E3 mice. (**C**)**.** In young E3 mice, freezing levels were significantly lower on days 2–4 than on day 1. ** *p* = 0.0012, **** *p* < 0.0001 versus day 1 (Dunnett’s). (**D**). In middle-aged E3 mice, freezing levels were significantly lower on days 3 and 4 than day 1. * *p* = 0.047, ** *p* = 0.007 versus day 1 (Dunnett’s). (**F**). In middle-aged E4 mice, freezing levels were significantly lower on days 3 and 4 than day 1. * *p* < 0.05.

When the percent freezing during the four days of extinction was analyzed, there was an effect of day (*F*(3,210) = 68.297, *p* < 0.001); age (*F*(1,210) = 50.023, *p* < 0.001); a day × genotype interaction (*F*(7,210) = 3.233, *p* = 0.041); a day × housing interaction (*F*(3,210) = 6.202, *p* < 0.001); a sex × housing interaction (*F*(1,210) = 3.850, *p* = 0.051); and a day × sex × housing × age interaction (*F*(3,210) = 3.466, *p* = 0.016) (Figure 2). Based on these interactions, we pursued the analyses described below.

Based on the effect of age and the day × sex × housing × age interaction over the first four extinction days, we also analyzed the data in the younger and older mice separately.

When the percent freezing during the four days of extinction was only analyzed in the young mice, there was an effect of day (*F*(3,79) = 33.186, *p* < 0.001); genotype (*F*(2,81) = 3.182, *p* = 0.047); a day × genotype interaction (*F*(6,160) = 3.378, *p* = 0.003); and a genotype × housing interaction (*F*(2,81) = 3.138, *p* = 0.049). The percent freezing of E2 and E4 mice on days 9, 30, and 51 are illustrated in Figure S4.

When the percent freezing during the four days of extinction was only analyzed in the older mice, there was an effect of day (*F*(1,129) = 72.289, *p* < 0.001); sex (*F*(1,1129) = 8.436, *p* = 0.004); genotype (*F*(2,129) = 3.378, *p* = 0.037); a day × genotype (*F*(2,129) = 7.206, *p* = 0.001) and a day × housing interaction (*F*(1,129) = 12.545, *p* < 0.001); and a trend towards a sex x housing interaction (*F*(1,129) = 2.962, *p* = 0.088).

#### 2.1.5. Analysis Broken Down by Housing

Based on the day × housing interaction, the sex × housing interaction, and the day × sex × housing × age interaction, we also analyzed the data of single- and group-housed mice separately. When the percent freezing during the four days of extinction was only analyzed in singly housed mice, there was an effect of day (*F*(3,102) = 55.913, *p* < 0.001), an effect of age (*F*(1,102) = 32.614, *p* < 0.001), an age × genotype interaction (*F*(2,102) = 3.544, *p* = 0.033), and a day × genotype interaction.

#### 2.1.6. Analysis Broken Down by Genotype in Group-Housed Mice

Based on the day × genotype interaction, we also analyzed the three genotypes in group-housed mice separately. When the percent freezing during the four days of extinction was analyzed in only the E2 mice, there was an effect of day (3,73) = 6.842, *p* < 0.001) and an effect of age (*F*(1,75) = 26.893, *p* < 0.001), with higher freezing levels in middle-aged than young E2 mice (Figure 2A,B). However, although there was an overall effect of day, when the extinction of the young and middle-aged E2 mice was analyzed separately, there was no effect of day, and freezing on extinction days 2–4 was not different from that on day 1.

When the percent freezing during the four days of extinction was analyzed in only the E3 mice, there was an effect of day (*F*(1,77) = 135.374, *p* < 0.001); an effect of housing (*F*(1,77) = 5.081, *p* = 0.027); an effect of age (*F*(1,77) = 14.673, *p* < 0.001); a day × housing interaction (*F*(1,77) = 2.146, *p* < 0.001); and a day × age interaction (*F*(1,77) = 6.520, *p* < 0.001) (Figure 2C,D). When the percent freezing during the four days of extinction was analyzed in only the young E3 mice, there was an effect of day (*F*(3,71) = 25.71, *p* < 0.001), and freezing on days 2, 3, and 4 was lower than that on day 1 (Dunnett’s). When the percent freezing during the four days of extinction was analyzed in only the middle-aged E3 mice, there was an effect of day (*F*(3,68) = 3.532, *p* = 0.0179), and the freezing on days 3 and 4 was lower than that on day 1 (Dunnett’s).

When the percent freezing during the four days of extinction was analyzed in only the E4 mice, there was an effect of day (*F*(1,58) = 344.724, *p* < 0.001); an effect of age (*F*(1,77) = 10.222, *p* = 0.02); a day × housing interaction (*F*(1,77) = 2.971, *p* = 0.033); and a day × sex × housing × age interaction (*F*(3,174) = 4.740, *p* = 0.003) (Figure 2E,F). When the percent freezing during the four days of extinction was analyzed in only the young E4 mice, there was no effect of day and no significant difference in the percent freezing on days 2, 3, or 4 compared to that on day 1. However, when the percent freezing during the four days of extinction was analyzed in only the middle-aged E4 mice, there was an effect of day (*F*(3,88) = 5.431, *p* = 0.0080) and the freezing levels on day 3 (*p* = 0.0470, Dunnett’s) and day 4 (*p* = 0.0356, Dunnett’s) were lower than those on day 1.

#### 2.1.7. Analysis Broken Down by Genotype in Single-Housed Mice

**Figure 3:**
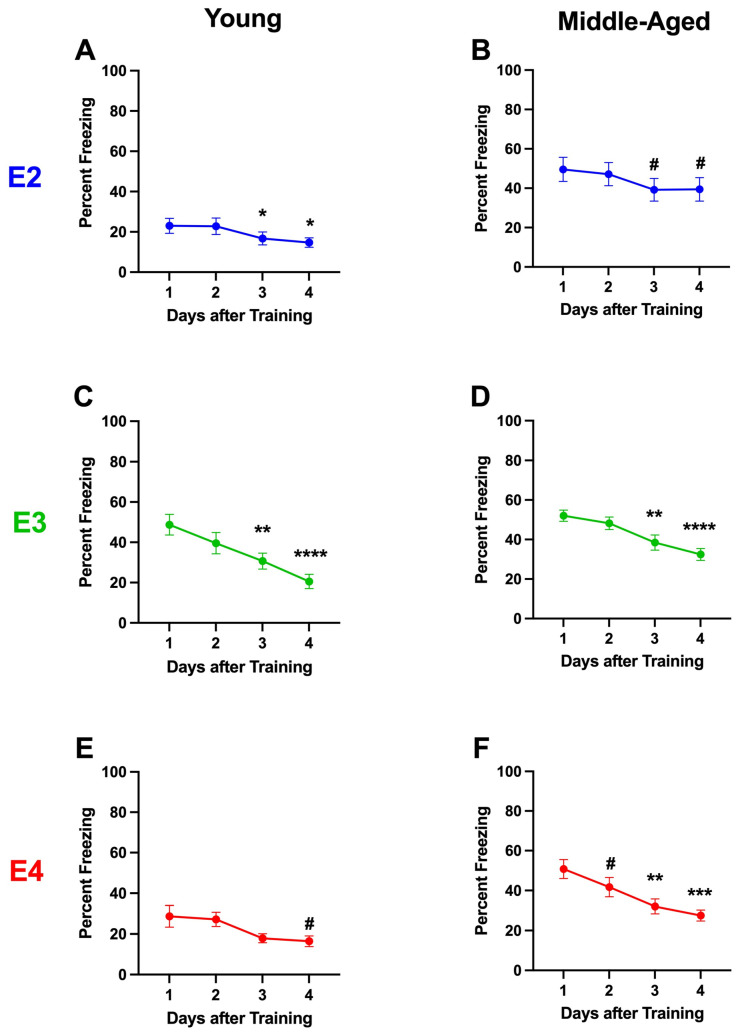
Extinction of contextual fear memory in young (**A**,**C**,**E**) and middle-aged (**B**,**D**,**F**) E2 (**A**,**B**), E3 (**C**,**D**), and E4 (**E**,**F**) single-housed mice. (**A**). In young single-housed E2 mice, there was an effect of day (*F*(3,83) = 5.486 *p* = 0.0050). * *p* < 0.05 versus day 1, Dunnett’s). (**B**). There was an effect of day in middle-aged E2 mice (*F*(3,67) = 5.108 *p* = 0.0132). There was a trend towards a lower percent freezing on days 3 (*p* = 0.0564, Dunnett’s) and 4 (*p* = 0.0915, Dunnett’s) than on day. (**C**). In young single-housed E3 mice, there was an effect of day (*F*(3,59) = 16.35 *p* < 0.0001). ** *p* = 0.0043, **** *p* < 0.001 versus day 1, Dunnett’s). (**D**). In middle-aged E3 mice, there was an effect of day (*F*(3,111) = 20.53, *p* < 0.0001). ** *p* = 0.0016, **** *p* < 0.001 versus day 1, Dunnett’s. (**E**). In single-housed young E4 mice, there was an effect of day (*F*(3,71) = 5.888, *p* = 0.0149). There was a trend towards a lower percent freezing on day 4 than day 1. ^#^ *p* = 0.0624, Dunnett’s. (**F**). In single-housed middle-aged E4 mice, there was an effect of day (*F*(3,59) = 10.92, *p* < 0.001. ** *p* = 0.0011, *** *p* = 0.0006 versus day 1, Dunnett’s). There was a trend towards a lower percent freezing on day 2 than day 1. ^#^ *p* = 0.0623, Dunnett’s.

We next analyzed the contextual fear extinction curves in single-housed mice. In contrast to young group-housed E2 mice, in young single-housed E2 mice, there was an effect of day (*F*(3,83) = 5.486, *p* = 0.0050), and the percent freezing on days 3 (*p* = 0.0234, Dunnett’s) and 4 (*p* = 0.011, Dunnett’s) was lower than that on day 1 (Figure 3A). In middle-aged E2 mice, there was an effect of day (*F*(3,67) = 5.108, *p* = 0.0132); there was a trend towards a lower percent freezing on days 3 (*p* = 0.0564, Dunnett’s) and 4 (*p* = 0.0915, Dunnett’s) than on day 1 (Figure 3B).

Like in group-housed young and middle-aged E3 mice, there was extinction of contextual fear memory in single-housed young and middle-aged E3 mice. In single-housed young E3 mice, there was an effect of day (*F*(3,59) = 16.35 *p* < 0.0001), and the percent freezing on days 3 (*p* = 0.0043, Dunnett’s) and 4 (*p* < 0.001, Dunnett’s) was lower than that on day 1 (Figure 3C). In middle-aged E3 mice, there also was an effect of day (*F*(3,111) = 20.53, *p* < 0.0001), and the percent freezing on days 3 (*p* = 0.0016 Dunnett’s) and 4 (*p* < 0.001, Dunnett’s) was lower than that on day 1 (Figure 3D).

In single-housed young E4 mice, there was an effect of day (*F*(3,71) = 5.888, *p* = 0.0149); there was a trend towards a lower percent freezing on day 4 than day 1 (*p* = 0.0624, Dunnett’s). In single-housed middle-aged E4 mice, there was an effect of day (*F*(3,59) = 10.92, *p* < 0.001); the percent freezing was lower on days 3 (*p* = 0.0011, Dunnett’s) and 4 (*p* = 0.0006, Dunnett’s) than day 1, and there was a trend towards a lower percent freezing on day 2 than day 1 (*p* = 0.0623, Dunnett’s).

### 2.2. Study 2

**Figure 4:**
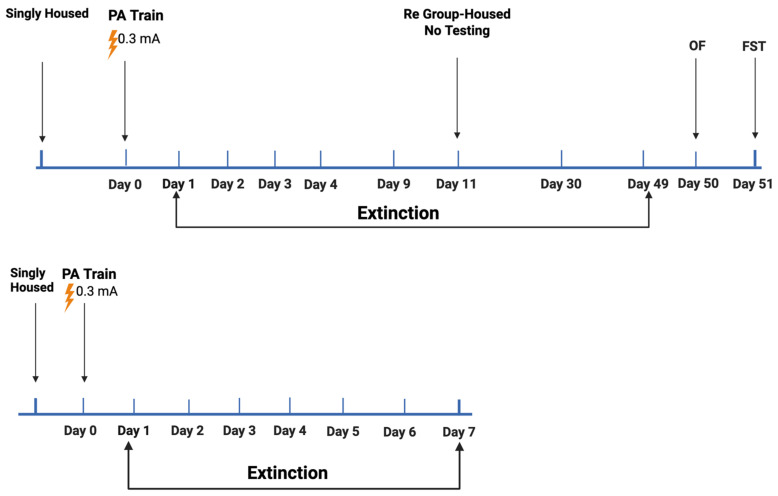
Experimental design of passive avoidance extinction followed by behavioral testing in the open-field and forced swim test in young (**Top**) and older (**Bottom**) mice. For details, see main text. Generated using Biorender. Bolis, M. (2025). Study 2: https://app.biorender.com/illustrations/65a1c40d895fa7d116cc1496?slideId=8565559e-9ded-430c-8d53-f4ca49427222 (accessed on 11 June 2025).

Study 2 (see Figure 4 for the experimental design) involved 135 mice (*n* = 70 male mice and *n* = 65 female mice; *n* = 42 E2 mice, *n* = 42 E3 mice, and *n* = 51 E4 mice; *n* = 83 young mice (3.69 ± 0.07 month of age) and *n* = 52 older mice (15.11 ± 0.34 months of age)). For the young mice (Figure 4 Top), as the latency to cross to the dark compartment did not show extinction within the four days, the mice were re-tested on day 9, group-housed after that, and re-tested on days 30 and 49, followed by open-field testing on day 50 and for performance in the forced swim test on day 51. The older mice were only tested for 7 days of passive avoidance extinction (Figure 4 Bottom).

When the latency to enter the dark compartment during passive avoidance training was analyzed, there were no effects or trends towards effects of sex, genotype, age, or interactions or trends towards interactions between them. These results indicate that passive avoidance learning was comparable across the groups.

When the passive avoidance memory extinction was analyzed during the first four days of extinction, there was an effect of day (*F*(1,108) = 18.965, *p* < 0.001); a day × genotype interaction (*F*(2,108) = 5.236, *p* < 0.001); a day × age × sex interaction (*F*(1,108) = 3.002, *p* = 0.031); and a day × age × genotype x sex interaction (*F*(2,108) = 3.026, *p* = 0.007).

Based on these statistical interactions, we next analyzed each extinction curve separately using a repeated-measures ANOVA and assessed whether the latency to cross into the dark compartment was different from that on day 1.

**Figure 5:**
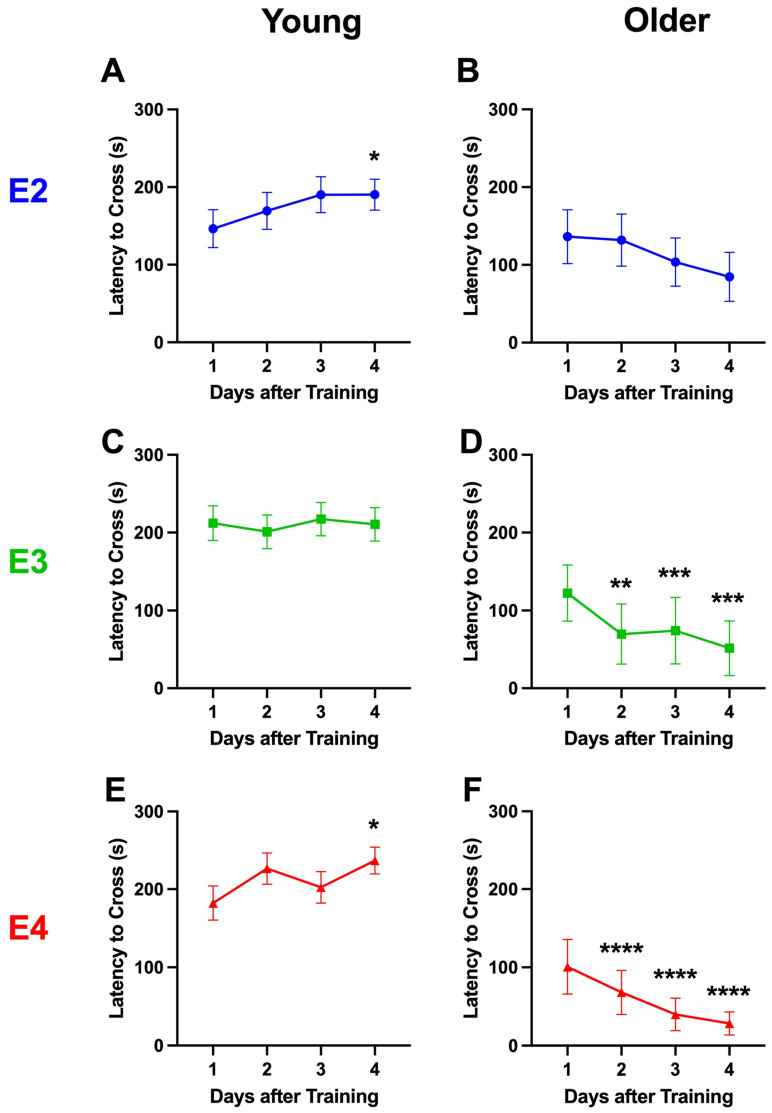
Latency to re-enter the dark compartment during extinction of passive avoidance memory in young (**A**,**C**,**E**) and older (**B**,**D**,**F**) E2 (**A**,**B**), E3 (**C**,**D**), and E4 (**E**,**F**) mice. (**A**). The latency of young E2 mice to re-enter the dark compartment. There was an effect of day (*F*(3,100) = 2.873, *p* = 0.04) and the mice re-entered the dark compartment slower on day 4 than day 1. * *p* = 0.0320 (Dunnett’s). (**B**). The latency of older E2 mice to re-enter the dark compartment. There was no effect of day on the latency to re-enter the dark compartment. (**C**). The latency of young E3 mice to re-enter the dark compartment. There was no effect of day on the latency to re-enter the dark compartment. (**D**). The latency of older E3 mice to re-enter the dark compartment. There was an effect of day (*F*(3,21) = 8.237, *p* = 0.0003) and the mice re-entered the dark compartment faster on days 2–4 than on day 1. ** *p* = 0.0022 versus day 1 (Dunnett’s). *** *p* < 0.001 versus day 1 (Dunnett’s). (**E**). The latency of young E4 mice to re-enter the dark compartment. There was an effect of day (*F*(3,112) = 3.395, *p* = 0.0204) and the mice re-entered the dark compartment slower on day 4 than day 1. * *p* = 0.0174 (Dunnett’s). (**F**). The latency of older E4 mice to re-enter the dark compartment. There was an effect of day (*F*(3,44) = 7.233, *p* = 0.0005) and the mice re-entered the dark compartment faster on days 2–4 than day 1. **** *p* < 0.0001 versus day 1 (Dunnett’s).

In young E2 (Figure 5A), E3 (Figure 5C), and E4 (Figure 5E) mice, there was no extinction of passive avoidance memory. Actually, in young E2 and E4 mice, the latency to re-enter the dark compartment increased over the days, and the mice re-entered the dark compartment slower on day 4 than day 1 (Figure 5A,E). In young E3 mice, the latency to re-enter the dark compartment was comparable across days (Figure 5C). For the extinction curves of the young mice including days 9, 30, and 49, see Figure S5. However, in older E3 (Figure 5D) and E4 (Figure 5F) mice, there was extinction of passive avoidance memory, and the mice re-entered the dark compartment faster on days 2–4 than on day 1. In contrast, no extinction was seen in older E2 mice (Figure 5B).

**Figure 6:**
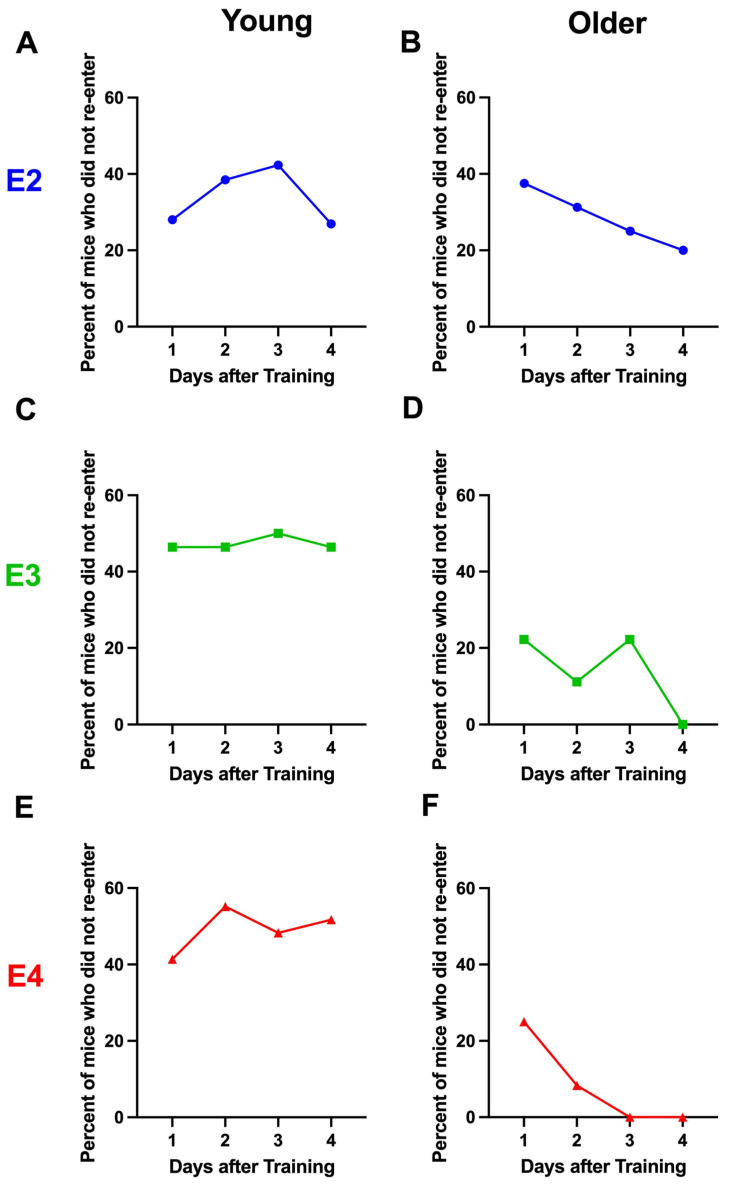
Percent of mice who did not re-enter the dark compartment during extinction of passive avoidance memory in young (**A**,**C**,**E**) and older (**B**,**D**,**F**) E2 (**A**,**B**), E3 (**C**,**D**), and E4 (**E**,**F**) mice.

We next analyzed the percentage of mice that did not re-enter the dark compartment. In the young mice, there was no decrease in the percentage of mice that did not re-enter the dark compartment, and on day 4, 25% of the young E2 (Figure 6A) and over 40% of the young E3 (Figure 6C) and E4 (Figure 6E) mice did not re-enter the dark compartment. Consistent with the older E3 and E4 mice being the only groups to show extinction of passive avoidance memory, older E3 and E4 mice were also the only groups with all mice re-entering on day 4 Figure 6D) and days 3 and 4 (Figure 6F), respectively. In contrast to older E3 and E4 mice, while the percentage of older E2 mice who did not re-enter the dark compartment decreased by about 50% over the extinction days, not all mice re-entered the compartment on day 4. This might be related to the fact that on day 1, the percentage of mice that did not re-enter was about twice as high in older E2 than older E3 and E4 mice.

### 2.3. Study 3

**Figure 7:**
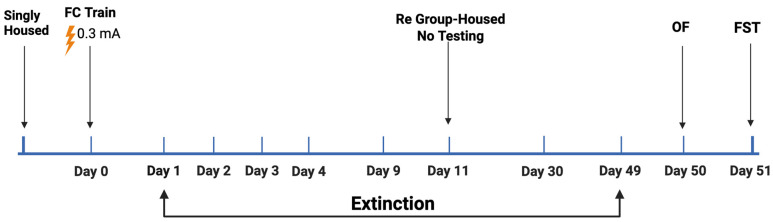
Experimental design of contextual fear extinction followed by behavioral testing in the open-field and forced swim test in young mice following training involving one shock. For details, see main text. Generated using Biorender. Bolis, M. (2025). Study 3: https://app.biorender.com/illustrations/657cd25a3dd5d75a184f7874?slideId=fdbe3221-ae37-4fd6-b8aa-758347bcf4ed (accessed on 11 June 2025).

In a separate cohort of young mice (*n* = 12 male mice; *n* = 4 male mice/genotype; age: 4.8 ± 0.21 months of age) (see Figure 7 for the experimental design), we matched the shock intensity and training trial time window used in the passive avoidance test (0.3 mA, see below) in the fear conditioning test. On training day, following a 20 s baseline period, the mice received a single shock (0.3 mA, 2 s). The total test trial lasted 30 s. The mice were put back in the chamber for 300 s on days 1, 2, 3, 4, and 9 following training and the percent freezing was analyzed. The mice were group-housed the following day and re-tested in the fear conditioning or passive avoidance test on day 49. On day 50, the mice were tested for measures of activity and anxiety in the open field, and on day 51 for depressive-like behavior in the forced swim test.

**Figure 8:**
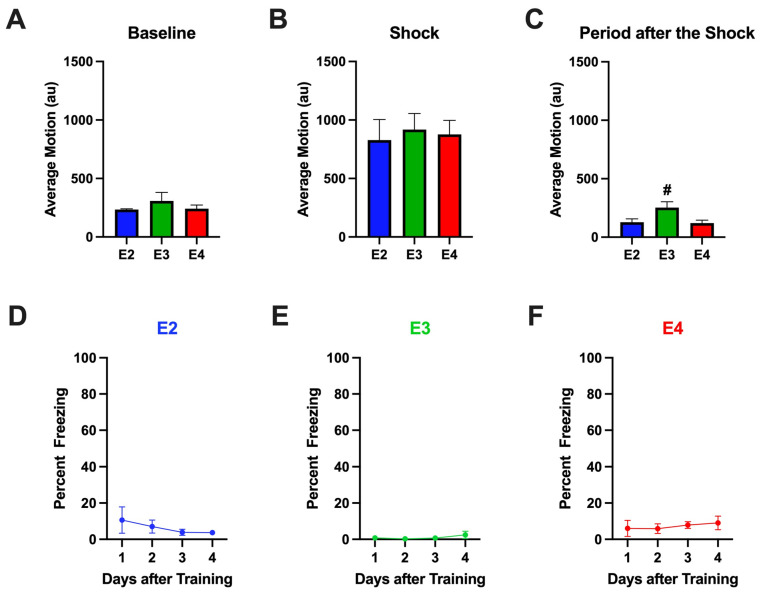
(**A**). There was no effect of genotype on activity levels during the baseline period in Study 3. (**B**). There was no effect of genotype on the response to the shock in Study 3. (**C**). There was a trend towards an effect of genotype on activity levels in the period after the shock (*F* = 4.145, *p* = 0.0530). There was a trend towards higher activity levels in E3 than E2 or E4 mice. # *p* = 0.0888 versus E2 and *p* = 0.0718 versus E4, Dunnett’s. (**D**). Percent freezing of E2 mice during the four days after training. (**E**). Percent freezing of E3 mice during the four days after training. (**F**). Percent freezing of E4 mice during the four days after training.

We next matched the shock intensity and limited the number of shocks to one in the fear conditioning test to match the training conditions in the passive avoidance test. There was no effect of genotype on activity levels during the baseline period (Figure 8A) or motion in response to the shock (Figure 8B). There was a trend towards an effect of genotype on activity levels following the shock (*F* = 4.145, *p* = 0.0530), with a trend towards higher activity levels in E3 than E2 (*p* = 0.0888) or E4 (*p* = 0.0718, Dunnett’s) mice (Figure 8C). There was minimal freezing during the training under these conditions. Only 25% of the E2 and E4 and none of the E3 mice showed freezing during the post-shock period on the training day under these experimental conditions. None of the mice showed freezing during the baseline period.

When the extinction of contextual fear was analyzed over the first four days of extinction, the percent freezing was very low (Figure 8D–F). There was a day × genotype interaction (*F*(3,27) = 4.541, *p* = 0.011). Based on the day × genotype interaction, we analyzed the genotypes separately. When the extinction of contextual fear was analyzed over the first four days of extinction in E2, E3, or E4 mice only, there were no significant effects. Based on this result, we next analyzed whether there was an effect of genotype in any of the four days of extinction. There was no effect of day in any of the three genotypes but there was an effect of genotype on only day 3 (*F* = 6.384, *p* = 0.0188), with a higher percent freezing in E4 (Figure 8F) than E3 (Figure 8E) mice (*p* = 0.0151, Tukey’s). There was also a trend towards an effect of genotype (*F*(1,27) = 23.502, *p* = 0.073), with a lower percent freezing in E3 than E2 or E4 mice. For the percent freezing of these mice including days 9, 30, and 49, see Figure S6.

#### 2.3.1. Comparison of Behavioral Performance in the Open-Field and Forced Swim Tests in Mice Tested for Passive Avoidance (Study 2) or Fear Conditioning (Study 3)

**Figure 9:**
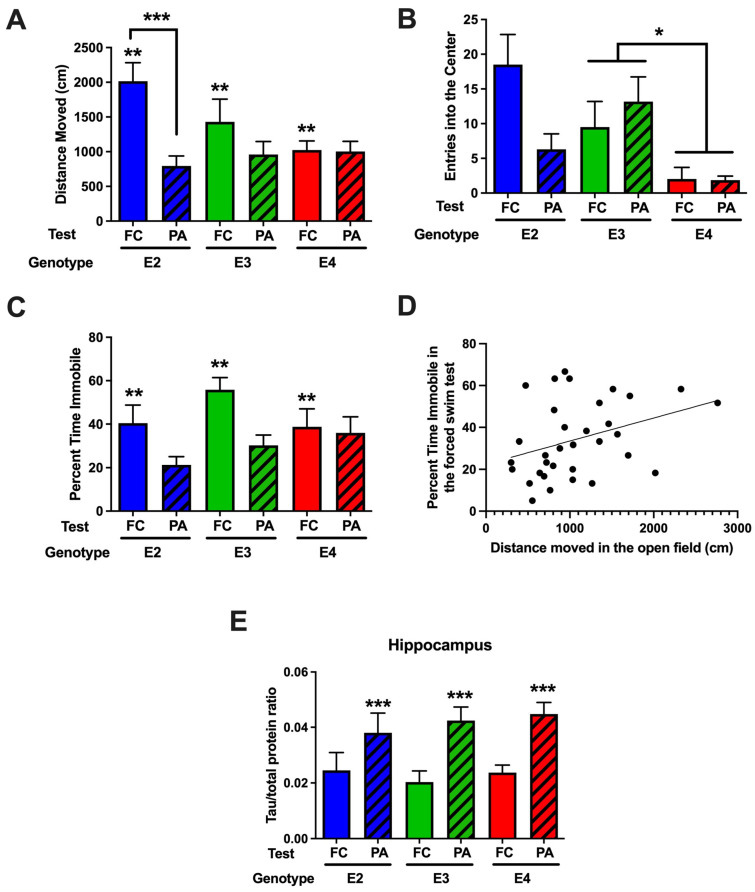
(**A**). Activity levels in the open field following fear conditioning (FC) and passive avoidance (PA) testing. Activity levels were lower in mice tested for passive avoidance than fear conditioning extinction. Effect of test (FC versus PA), ** *p* = 0.003. There was a genotype x study interaction (*F*(2,39) = 3.811, *p* = 0.032); in E2 mice, activity levels were lower in mice who received PA extinction testing than those who received FC extinction testing, *** *p* = 0.0010, *t*-test. (**B**). E4 mice entered the center less than E3 mice. * *p* = 0.0128, Tukey. (**C**). The percent time spent immobile was higher in mice tested for fear conditioning than for passive avoidance extinction. Effect of test (FC versus PA), ** *p* = 0.005. *n* = 14 E2 mice, *n* = 15 E3 mice, and *n* = 11 E4 mice. (**D**). The percent time spent immobile in the forced swim test was positively correlated with the activity levels in the open field. *r* = 0.3719, *p* = 0.0331 (Spearman, *n* = 33 data points). (**E**). Hippocampal tau levels in mice tested for FC and PA. Hippocampal tau levels were higher in mice tested for passive avoidance than those tested for fear conditioning. *** *p* < 0.0001. *n* = 10 E2 mice, *n* = 10 E3 mice, and *n* = 9 E4 mice.

When activity levels in the open field were analyzed, there was an effect of study (*F*(1,39) = 10.252, *p* = 0.003) and a genotype × study interaction (*F*(2,39) = 3.811, *p* = 0.032) (Figure 9A). Activity levels were lower in mice tested for passive avoidance than fear conditioning extinction.

When entries into the more anxiety-provoking center of the open field were analyzed, there was an effect of genotype (*F*(2,39) = 5.238, *p* = 0.010). E4 mice entered the center less than E3 mice (*p* = 0.0128, Tukey), and there was a trend of E4 mice to enter the center less than E2 mice (*p* = 0.073, Tukey). There was also a trend towards a study × genotype interaction (*F*(2,39) = 2.926, *p* = 0.067) (Figure 9B). There was no effect of study, genotype, or study × genotype interaction for time spent in the center of the open field.

When depressive-like behavior in the forced swim test was analyzed, there was an effect of study (*F*(1,39) = 8.829, *p* = 0.005) (Figure 9C). The percent time spent immobile was higher in mice tested for fear conditioning than for passive avoidance extinction. Based on the similar pattern between the activity levels in the open field and percent time spent immobile in the forced swim test, we assessed whether these distinct behavioral measures were correlated. The percent time spent immobile in the forced swim test was positively correlated with the activity levels in the open field (*r* = 0.3719, *p* = 0.0331, Spearman, *n* = 33 data points, Figure 9D).

#### 2.3.2. Comparison of Hippocampal MAP2, Syn, and Tau Levels in Mice Tested for Passive Avoidance (Study 2) or Fear Conditioning (Study 3)

There was no difference in hippocampal Syn (*t* = 1.616, *p* = 0.1163, Figure S7A) or MAP2 (*t* = 1.616, *p* = 0.1163, Figure S7B) levels in mice tested for passive avoidance and fear conditioning. There was no genotype difference in hippocampal Syn (Figure S7C) or MAP2 (Figure S7D) levels either. However, when hippocampal tau levels were analyzed, they were higher in mice tested for passive avoidance than those tested for fear conditioning (*F*(1,33) = 14.281, *p* < 0.001) (Figure 9E), while there were no genotype difference in mice tested for passive avoidance or fear conditioning.

## 3. Discussion

The behavioral results of the studies are summarized in Table 1. In young group-housed mice, E3, but not E2 or E4, mice showed extinction of contextual fear memory. In middle-aged group-housed mice, E3 and E4, but not E2, mice showed extinction of contextual fear memory. Compared to group-housed mice, more extinction of contextual fear memory was seen in single-housed mice. In young single-housed mice, E2 and E3, but not E4, mice showed extinction of contextual fear memory. In middle-aged single-housed mice, E3 and E4, but not E2, mice showed extinction of contextual fear memory. Thus, with regard to the extinction of contextual fear memory, E2 and, to a lesser extent, E4 mice showed impaired extinction. In contrast to the extinction of contextual fear memory, no extinction of passive avoidance memory was seen in young mice of any genotype. In older mice, extinction of passive avoidance memory resembled the pattern seen for extinction of contextual fear memory: extinction of passive avoidance memory in E3 and E4, but not E2, mice. The lack of extinction of passive avoidance memories in the young E2, E3, and E4 mice was profound and long-lasting; compared to day 9, extinction was seen in E2 and E4 mice on days 30 and 49 and in E3 mice on day 49 (Figure S5). In contrast, in older E3 and E4 mice extinction of passive avoidance memory was already seen on day 2. It is conceivable that the enhanced avoidance seen in younger mice is due to enhanced traumatic memories. These data are consistent with the association of E2 [26,27,28] and E4 [29,30,31] with the development of PTSD. Future studies are warranted to determine what pathways in E3 mice that are not present in E2 and E4 mice might contribute to the enhanced extinction of contextual fear and passive avoidance memories. Especially considering that the contextual fear training in Studies 1 and 2 involved two shocks of 0.5 mA, while the passive avoidance training involved one shock of 0.3 mA, these data highlight the importance of considering avoidance behavior in animal models of PTSD. Consistent with this, hardly any freezing was seen during extinction of contextual fear trials when the training only involved a matching single shock of 0.3 mA. The relatively strong passive avoidance memory compared to contextual fear memory was associated with reduced activity levels in the open field and decreased time spent immobile in the forced swim test, especially in E2 mice, and with increased hippocampal tau levels in all genotypes. As passive avoidance memories were comparable in young E2 and E4 mice, these data suggest that E2 mice might be more sensitive than E4 mice to the impact of comparable avoidance memories on exploratory behavior and depressive-like behavior.

The pattern of activity levels in the open field and of percent immobility in the forced swim test was similar, and a positive correlation was seen between these distinct behavioral measures. This result is remarkable as activity levels in the open field are typically not considered to be related to depressive-like behaviors [59] and rats who show higher activity levels in a novel environment show reduced immobility in the forced swim test [60]. The results of the current study suggest that it might be hard to assess depressive-like behavior in mice in the open field. This is important to recognize as there is increasing pressure to replace the forced swim test [61].

In comparing extinction of group versus single-housed mice, group-housed E3 mice started to show extinction on the second extinction day, while singly housed E3 mice started to show extinction on the third extinction day. This result is consistent with empathy facilitating extinction. However, in contrast, while young singly housed E2 mice showed extinction on days 3 and 4, no extinction was seen in young group-housed E2 mice. In addition, no significant differences were seen between the extinction of group and single-housed E4 mice. Together, these data suggest that empathy or social buffering does not necessarily alter extinction of contextual fear memory when animals are tested individually. Consistent with this result, while reduced fear responses during extinction were seen in paired tested rats, this effect was transient and did not remain when the animals were tested individually, independent of whether or not they were paired prior to the extinction testing [62]. While it is possible that empathy might be apoE isoform-dependent, no difference in facial emotion recognition were seen in patients with subjective cognitive decline, mild cognitive impairment, or AD [63].

The current study involved conventional targeted replacement mice. Future studies are warranted to use methodologies allowing a greater anatomical and temporal precision to investigate the role of distinct apoE isoforms in specific brain regions, such as the hippocampus, and in specific cell types in specific brain regions, and at different time points.

From the three hippocampal protein markers we analyzed, tau levels were about two times higher in mice that received passive avoidance memory extinction than contextual fear extinction. The elevated tau levels might relate to trauma and PTSD and tau might be a biomarker linking PTSD and AD [64]. Plasma levels of tau were higher in military personnel with a history of mild traumatic brain injury and PTSD than those with mild traumatic brain injury without PTSD and those without mild traumatic brain injury or PTSD [65]. In addition, in patients with mild traumatic brain injury, plasma tau levels were elevated and correlated with clinical variable of trauma severity [66], and in athletes, acutely elevated plasma tau levels correlated with return to play [67]. Plasma tau levels were also elevated in responders to the World Trade Center attack compared to controls but were not different between responders who did or did not develop PTSD [68]. Consistent with that observation, in Vietnam Veterans, PTSD was not associated with altered levels of brain or cerebrospinal tau levels [69]. Similarly, in people 50 age and older, serum tau levels were comparable in those with PTSD and those exposed to trauma who did not develop PTSD [70]. Consistent with these human data, hippocampal tau levels were increased following social defeat stress [55]. However, it is also possible that the elevated hippocampal tau levels in mice that received passive avoidance versus contextual fear memory extinction is not related to the experienced trauma but just related to the enhanced memory seen in the passive avoidance memory extinction paradigm compared to the contextual fear memory extinction paradigm. Hippocampal tau levels are involved in synaptic plasticity [53], spatial memory retention in the water maze, and rotarod performance [54]. Future efforts are warranted to assess alterations in hippocampal pathways in human apoE mice exposed to these two paradigms using unbiased omics analyses to ultimately improve the quality of life and care of those exposed to traumatic events.

## 4. Materials and Methods

**Figure 10:**
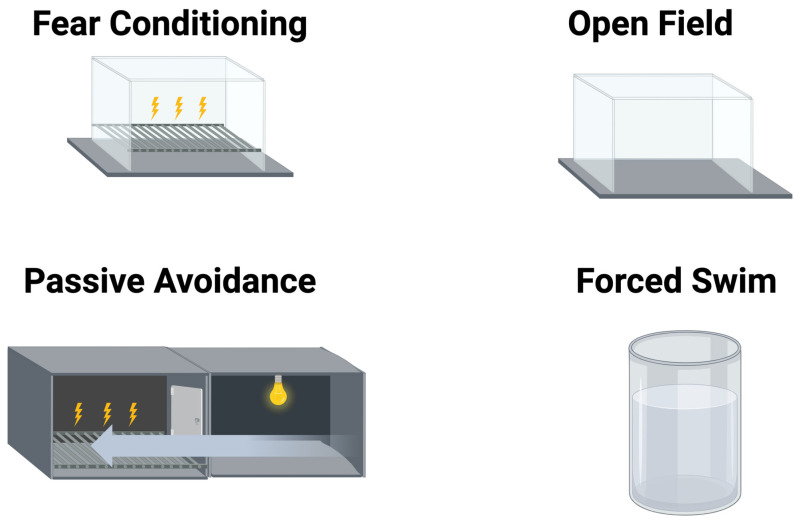
Illustrations of the behavioral tests. In the fear conditioning test (**top left**), the mice are trained and tested in a single enclosure. In the passive avoidance test (**bottom left**), the mice can choose out of two compartments: the dark compartment in which they received a shock before or the light compartment. In the open-field test (**top right**), measures of activity and anxiety are being assessed. In the forced swim test (**bottom right**), depressive-like behavior is being assessed. Generated using Biorender. Bolis, M. (2025). https://app.biorender.com/illustrations/66107c92aae432021fff877b (accessed on 11 June 2025).

The behavioral paradigms are illustrated in Figure 10 and described in detail below. All procedures complied with the National Institutes of Health Guide for the Care and Use of Laboratory Animals and with IACUC approval at Oregon Health & Sciences University. Experimenters were blinded to the genotype and sex of the mice.

### 4.1. Open-Field Test

The mice were tested in an open-field enclosure (16 × 16 inches, Kinder Scientific, Poway, CA, USA) for 10 min. Activity levels (distance moved) and time spent and entries into the more anxiety-provoking center (8 × 8 inches) of the open field were analyzed using Noldus Ethovision video tracking software (version 17, Wageningen, The Netherlands).

### 4.2. Forced Swim Test

To assess depressive-like behavior, mice were placed for 6 min in a container with water (water height: 15 cm; container diameter: 16–20 cm; 25 °C) not allowing the mouse’s tail to touch the bottom. Immobility, defined as cessation of limb movements except minor involuntary movements of the hind limbs or those movements necessary to stay afloat, was scored manually, by an observer blinded to genotype and test history using a sampling technique every 5 s during the trial. The data are expressed as the percentage immobility (number of immobility observations divided by the total number of observations) during the last 4 min (=48 observations) of the test, as described in [71].

### 4.3. Extinction of Passive Avoidance

The passive avoidance test was administered using a two-compartment chamber from Kinder Scientific (Poway, CA, USA). During the training day, the mice were placed in one compartment of the chamber. Both compartments were dark at first. After a brief habituation period (5 s), a cue light turned on and the connecting gate opened. Mice can escape the aversive light by entering the dark compartment. Upon entering, they receive a mild foot shock (0.3 mA, 2 s) and the time to cross into the dark chamber is recorded. On four subsequent days, the mice were put back in the light compartment, but no shock was delivered if they moved to the dark compartment. The trial ended if the mice entered the dark compartment within 300 s or at 300 s if the mice did not enter the dark compartment within this time period.

### 4.4. Microtubule-Associated Protein 2 (MAP2) and Synaptophysin (Syn) ELISAs

Following behavioral testing, the mice were euthanized by cervical dislocation without anesthesia. To determine whether hippocampal levels of MAP2 and Syn would show differences based on genotype and/or whether the mice were tested for extinction of contextual fear or passive avoidance memory, hippocampi from a subgroup of 4-month-old mice (E2: 4.24 ± 0.04 months; E3: 4.07 ± 0.25 months; and E4: 4.78 ± 0.31 months) who received 0.3 mA and a single shock during contextual fear learning (Study 3) and passive avoidance learning (Study 2) were processed for ELISAs as described [72] and analyzed using MyBioSource ELISAs (San Diego, CA, USA). For the ELISAs, the standard curve was run as duplicates and the samples as singles. The samples were examined for protein concentration by the Bradford assay (BioRad, Hercules, CA, USA) and were diluted with PBS for ELISA or dot blot analysis and stored at −80 °C until use.

### 4.5. Tau Dot Blot Analysis

The hippocampal samples described above were analyzed for tau protein levels by dot blot, as described [73]. Diluted samples (2.4 µg protein/well) were first applied on a 0.45 µM nitrocellulose membrane in a dot blot apparatus (BioRad, Hercules, CA, USA). The membranes were pre-wetted with TBS at RT and gently shaken for 10 min and assembled in the dot blot apparatus. The wells were filled with 100 mL of TBS and vacuum was used to pull the solution through. Subsequently, 10 μL of sample in PBS (without detergent) per well were applied. The vacuum was left off for an hour, followed by a gentle vacuum to remove the remaining liquid. Subsequently, the membrane was removed from the apparatus and allowed to air dry overnight.

The membrane was rinsed in MilliQ water for 5 min and the total protein content assessed by Totalstain Q (Azure Biosystems, Dublin, CA, USA). The membrane was incubated with Totalstain for 10 min, under gently shaking conditions. Subsequently, the membrane was washed three time for 3 min with 10 mL Working Washing Solution (Azure Biosystems) and imaged using an Azure600. Membranes were then blocked with LiCor Intercept^®^ (TBS) (Lincoln, NE, USA) blocking buffer for 60 min at RT and incubated overnight with the primary antibody (tau5: 1:1000, Invitrogen, Waltham, MA, USA, MA5-12808) ON at 40C while shaking. The membrane was washed 3 times for 10 min with TBST (TBS containing 0.1% Tween-20) and incubated with the secondary antibody (Goat anti mouse IgG (IRDye 800cw, Li-COR, Lincoln, NE, USA); 1:10,000) antibodies diluted in 50:50 Intercept^®^ Blocking Buffer (New York, NY, USA): TBS with 0.01% Tween-20 for 60 min in the dark at RT. After secondary antibody incubation, the membranes were washed 2 × 10 min in TBST and 1 × 15 min in TBS and imaged using the Azure600. Total protein levels were analyzed using Cy3 and tau protein levels using IRDye800cw. For each dot, data are expressed as (intensity of tau protein pixels (Cy3)/intensity/intensity of total protein pixels (IRDye800cw). Images of the samples are illustrated in Figure S8.

### 4.6. Statistical Analyses

All behavioral data are reported as mean ± standard error of the mean and were analyzed using SPSS v.22 (IBM, Armonk, NY, USA) or GraphPad v.10 (La Jolla, CA, USA) software.

For each of the studies, genotype, age, and sex were included as factors in analyses of variance (ANOVAs). For the young mice tested for extinction of contextual fear, housing condition during the first week was included as a factor in the analysis as well. In cases where there were statistical interactions, ages, genotypes, sexes, and housing conditions were analyzed separately, as indicated. Repeated measures were used when appropriate. We first analyzed the first four days of extinction learning. For those mice who received additional days of memory extinction training, we analyzed those additional extinction testing days separately. ELISA data were analyzed with the genotype and behavioral test used as factors in the analysis. Statistical significance was considered as *p* < 0.05. When sphericity was violated (Mauchly’s test), Greenhouse–Geisser corrections were used. All researchers were blinded to genotype and age and the code was only broken after the data were analyzed.

## Figures and Tables

**Table 1:** Summary of contextual fear and passive avoidance memory extinction data.

***Contextual Fear Memory***	E2	E3	E4
Study 1, young group-housed mice		Extinction	
Study 1, middle-aged group-housed mice		Extinction	Extinction
Study 1, young single-housed mice	Extinction	Extinction	
Study 1, middle-aged single-housed mice		Extinction	Extinction
***Passive avoidance memory***	E2	E3	E4
Study 2, young mice			
Study 2, older mice		Extinction	Extinction

## Data Availability

All data is included with this study.

## Supplementary Materials

The following supporting information can be downloaded at https://www.mdpi.com/article/10.3390/ijms26125820/s1.
