# Supplementary material for: ApoE Isoform-Dependent Effects on Extinction of Contextual Fear Memory and Passive Avoidance Memory
Source: Int J Mol Sci. 2025 Jun 17;26(12):5820. doi: 10.3390/ijms26125820 (PMC12193006; doi:10.3390/ijms26125820)
Supplement: Supplementary file 1 [file ijms-26-05820-s001.zip › ijms-3656307-supplementary.pdf]

Supplementary Data and Figures

Study 1

Baseline activity levels during fear learning

For activity levels during the baseline period, there was a trend towards a genotype x sex interaction ($F(2,210) = 2.688$, $p = 0.070$), and a trend toward genotype x age x sex interaction ($F(2,210) = 2.540$, $p = 0.081$) (Fig. S1A).

Percent freezing during the ISIs during fear learning

When the percent freezing during the two ISIs was analyzed, there was a trend towards an ISI x sex interaction $F(1,210) = 3.837$, $p = 0.051$), a trend towards a housing x genotype interaction ($F(2,210) = 2.533$, $p = 0.082$), and a trend towards a housing x age interaction ($F(1,210) = 2.755$, $p = 0.098$) (Fig. S3).

Percent freezing during the extinction days

When the percent freezing during the four days of extinction was analyzed, there was a trend towards a day x sex x genotype x age interaction ($F(3,210) = 1.955$, $p = 0.070$).

When the percent freezing during the four days of extinction was only analyzed in the young mice, there was a trend towards a day x sex interaction ($F(2,129) = 2.524$, $p = 0.064$).

When the percent freezing during the four days of extinction was only analyzed in the older mice, there was a trend towards a sex x housing interaction ($F(1,129) = 2.962$, $p = 0.088$).

Analysis broken down by housing

When the percent freezing during the four days of extinction was only analyzed in singly housed mice, there was a trend towards an effect of genotype ($F(2,102) = 2.905$, $p = 0.059$), a trend towards a day x sex interaction.

Analysis broken down by genotype in group housed mice

When the percent freezing during the four days of extinction in only the E4 mice was analyzed, there was a trend towards a day x sex x age interaction ($F(3,174) = 2.340$, $p = 0.075$).

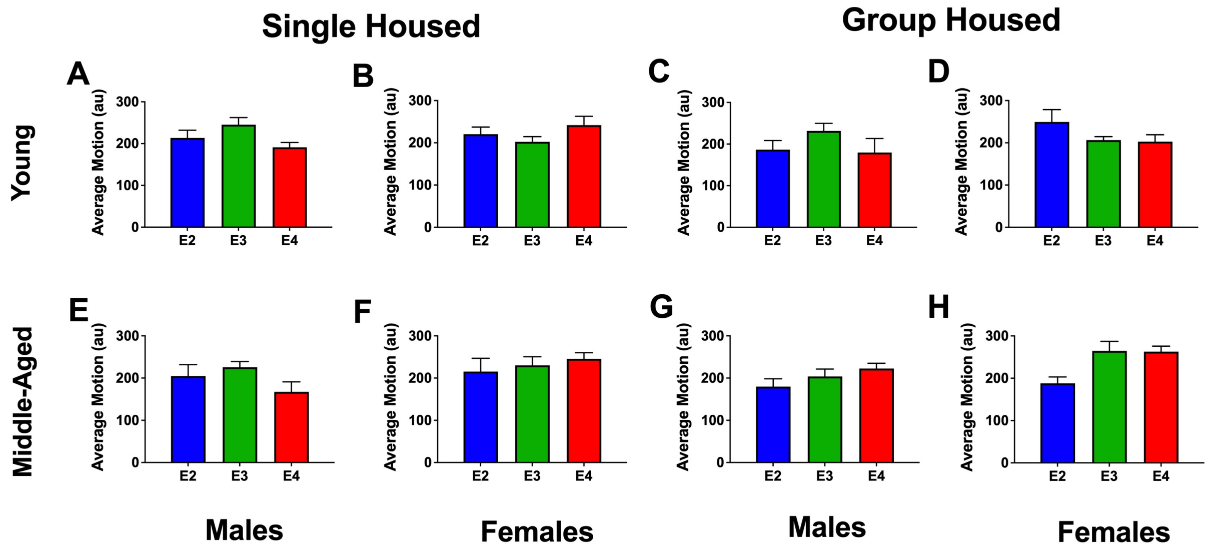

Fig. S1. The activity levels during the baseline period (*i.e.*, prior to the first shock during training) of single (**A, B, E, F**) and group (**C, D, G, H**) housed young (**A-D**) and middle aged (**E-H**) male (**A, C, E, G**) and female (**B, D, F, H**) mice in Study 1. There was an effect of sex ($F(1,210) = 7.272$, $p = 0.008$), with higher activity levels in females than males.

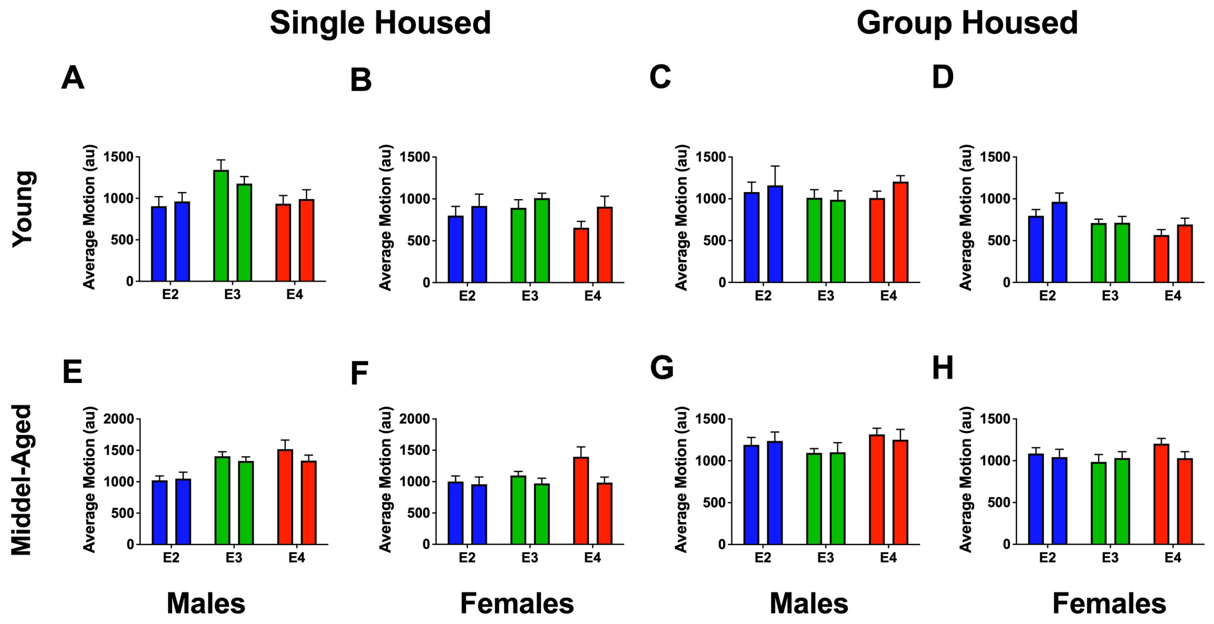

The two bars reflect the response to the two subsequent shocks. There was an effect of sex ($F(1,210) = 33.514$, $p < 0.001$), with a stronger response in males than females, and of age

($F(1,210) = 33.913$, $p < 0.001$), with a stronger response in young than middle-aged mice.**Fig. S2.** The response to the shocks of single (**A, B, E, F**) and group (**C, D, G, H**) housed young (**A-D**) and middle aged (**E-H**) male (**A, C, E, G**) and female (**B, D, F, H**) mice in Study 1.

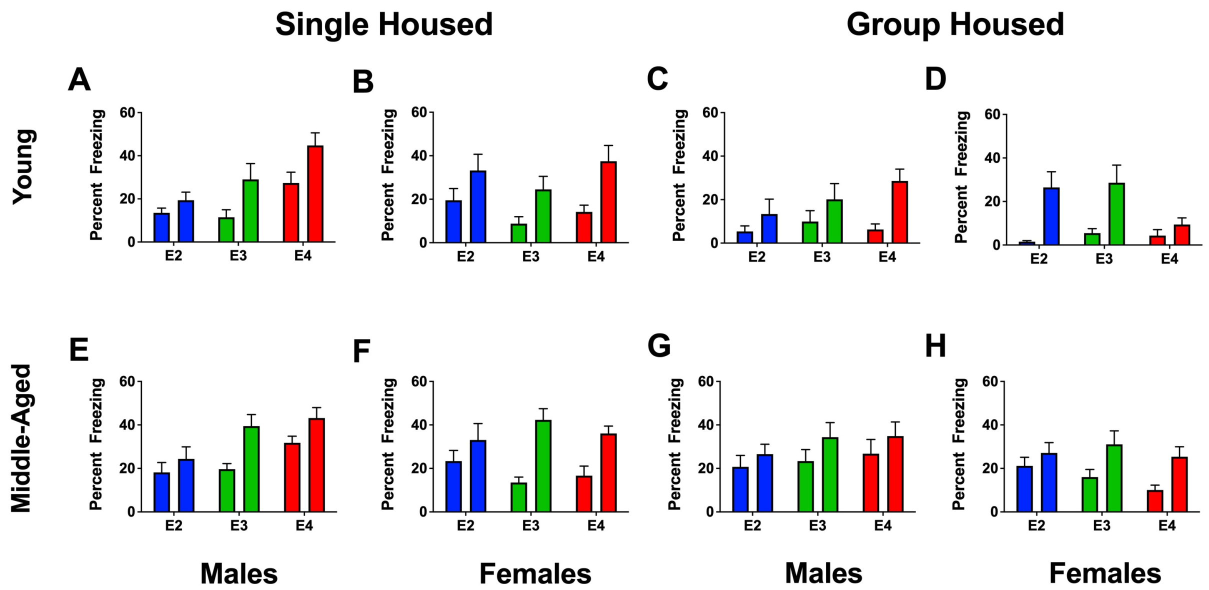

Fig. S3. Percent freezing during the ISIs of single (**A, B, E, F**) and group (**C, D, G, H**) housed young (**A-D**) and middle aged (**E-H**) male (**A, C, E, G**) and female (**B, D, F, H**) mice in Study 1.

A/C/E/G.

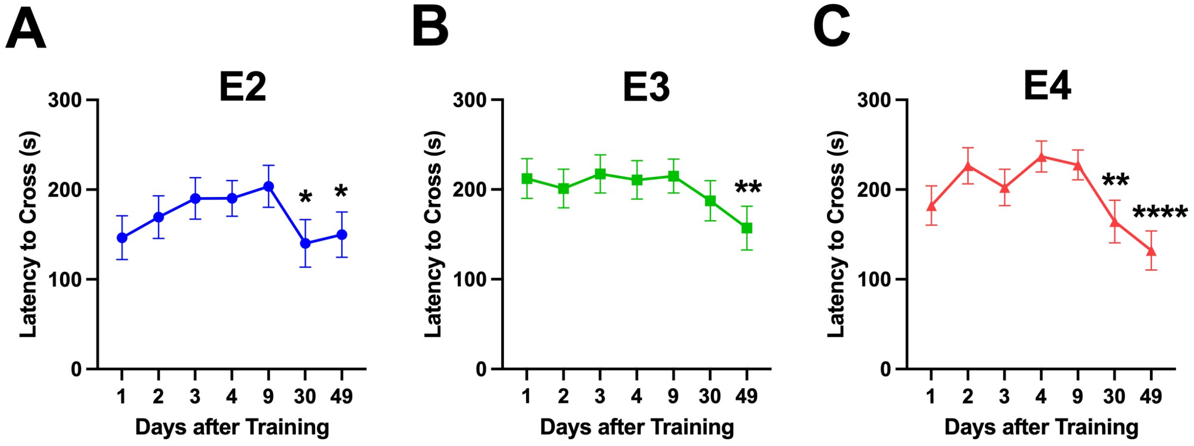

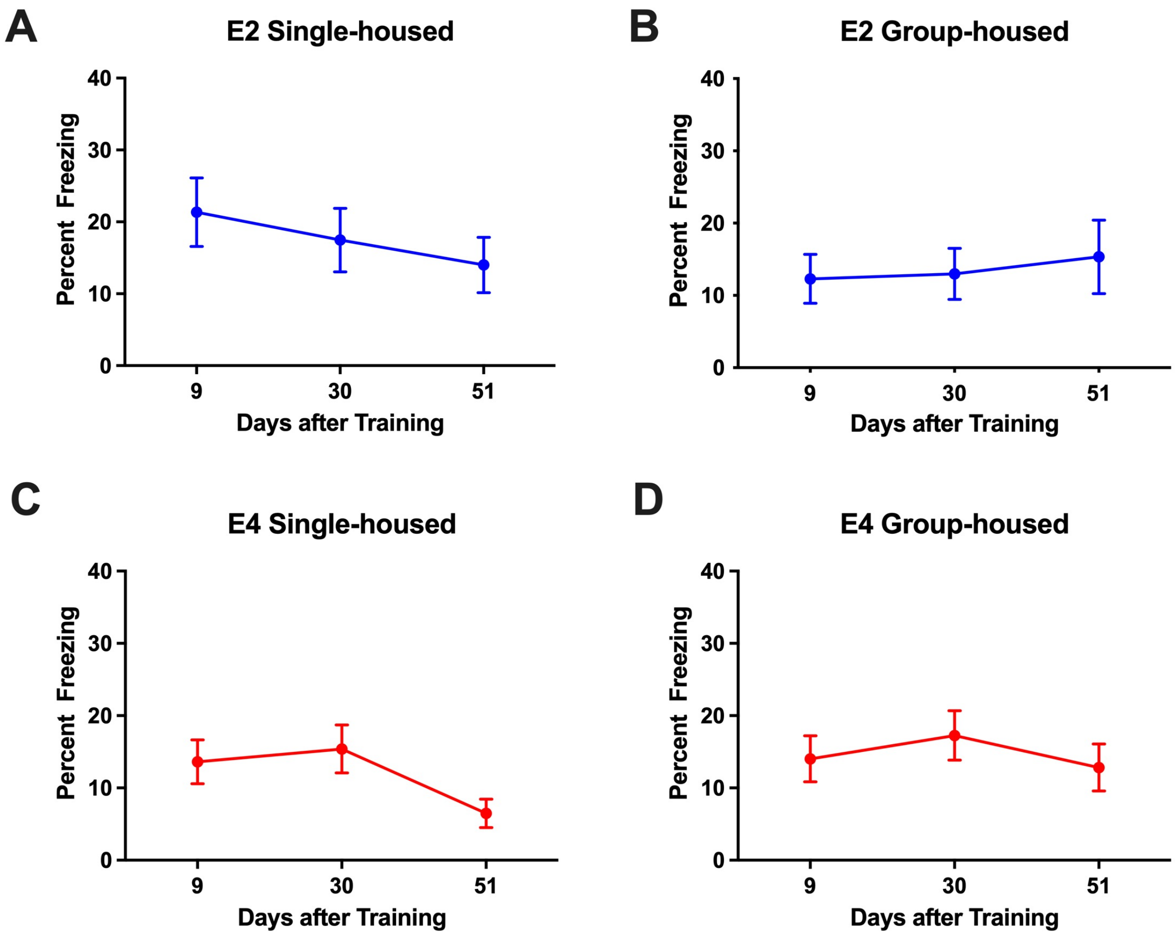

Fig. S4. Percent freezing of single (**A, C**) and group (**B, D**) young E2 (**A, B**) and E4 mice on days 9, 30, and 51 days after training. When all four groups were analyzed together, there was no effect of day, genotype, or housing. When each of the four groups was analyzed separately, there was only an effect of day in single-housed E4 mice ($F(1.626, 9.755) = 5.740$, $p = 0.0268$), but the percent freezing on days 30 and 51 was not different from that on day 9.

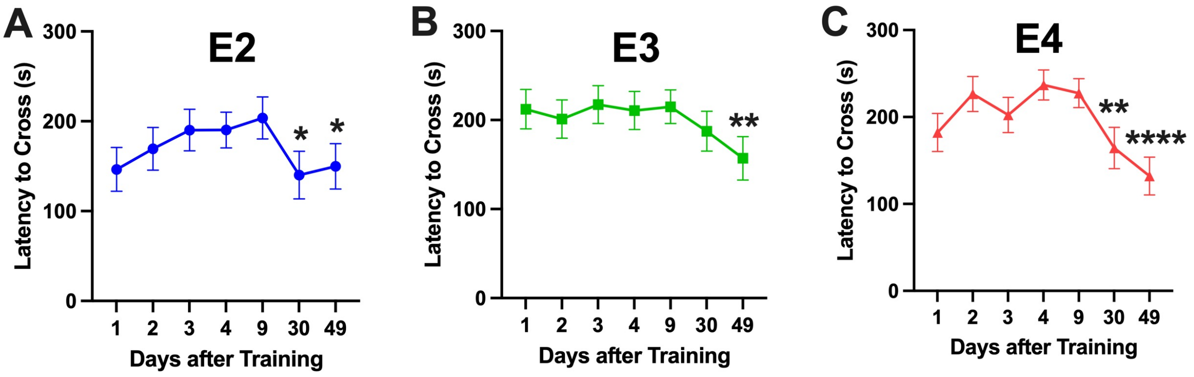

Fig. S5. Latency to re-enter the dark compartment during extinction of passive avoidance memory in young E2 (**A**), E3 (**B**), and E4 (**C**) mice. When all three genotypes were analyzed together, there was an effect of day $F(4.032, 298.350) = 12.157$, $p < 0.001$ (Greenhouse-Geisser) but no genotype difference. When each genotype was analyzed separately, there was an effect of day in E2 ($F(3.378,83.90) = 2.937$, $p = 0.0324$), E3 ($F(3.171,84.57) = 2.981$, $p = 0.0333$), and E4 ($F(4,079,114.2) = 7.981$, $p < 0.0001$) mice but days 2, 3, 4, 9, 30, and 49 were not different from day 1. As the latency seemed lower on days 30 and 49 than on day 9, we also analyzed only those three days. There was an effect of day ($F(2,150) = 23.766$, $p < 0.001$) but no overall genotype difference. There was an effect of day in E2 mice ($F(1.868,46.71) = 5.356$, $p = 0.0324$), with a lower latency on day 30 ($p = 0.0198$) and 49 ($p = 0.0336$) than day 9. There was an effect of day in E3 mice ($F(1.648,42.84) = 5.635$, $p = 0.01$), with a lower latency on day 49 ($p = 0.0024$) than day 9. There was an effect of day in E4 mice ($F(1.952,54.65) = 13.29$, $p < 0.0001$), with a lower latency on day 30 ($p = 0.0076$) and 49 ($p < 0.0001$) than day 9. *$p < 0.05$; **$p < 0.01$; ****$p < 0.0001$.

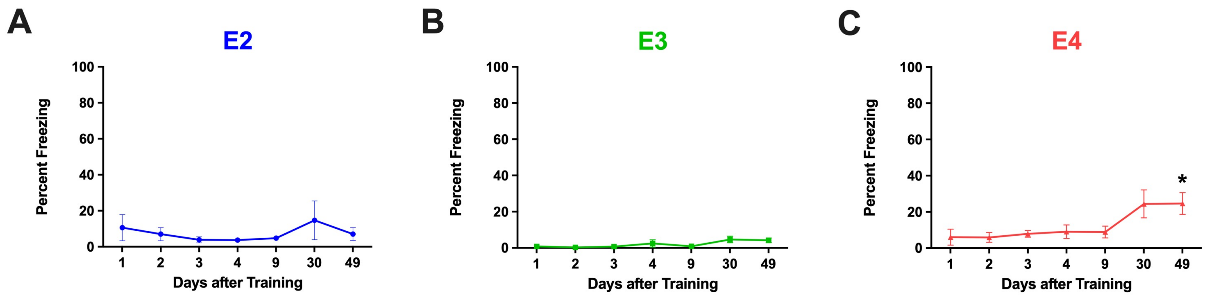

Fig. S6. Percent freezing of E2 (**A**), E3 (**B**), and E4 (**C**) mice in Study 3. When all three curves were analyzed together, there was an effect of day $F(1.682, 15.139) = 5.925$, $p = 0.016$) but no effect of genotype. When the curves were analyzed separately, there was no effect of day in E2 or E3 mice. In E4 mice, there was an effect of day (F(1.460, 4.379) = 11.83, p = 0.0195), with the percent freezing on day 49 being higher than that on day 1.*$p = 0.0122$. There was a trend towards higher freezing on day 30 than day 1 but it did not reach significance ($p = 0.0613$). When only days 9, 30, and 49 were analyzed, there was also no effect of day in E2 or E3 mice but there was an effect of day in E4 mice ($F(1.346, 4.037) = 9.777$, $p = 0.0319$), with a higher percent freezing on day 49 than day 9. *$p = 0.0268$. There was a trend towards a higher percent freezing on day 30 than day 9 but it did not reach significance ($p = 0.0992$).

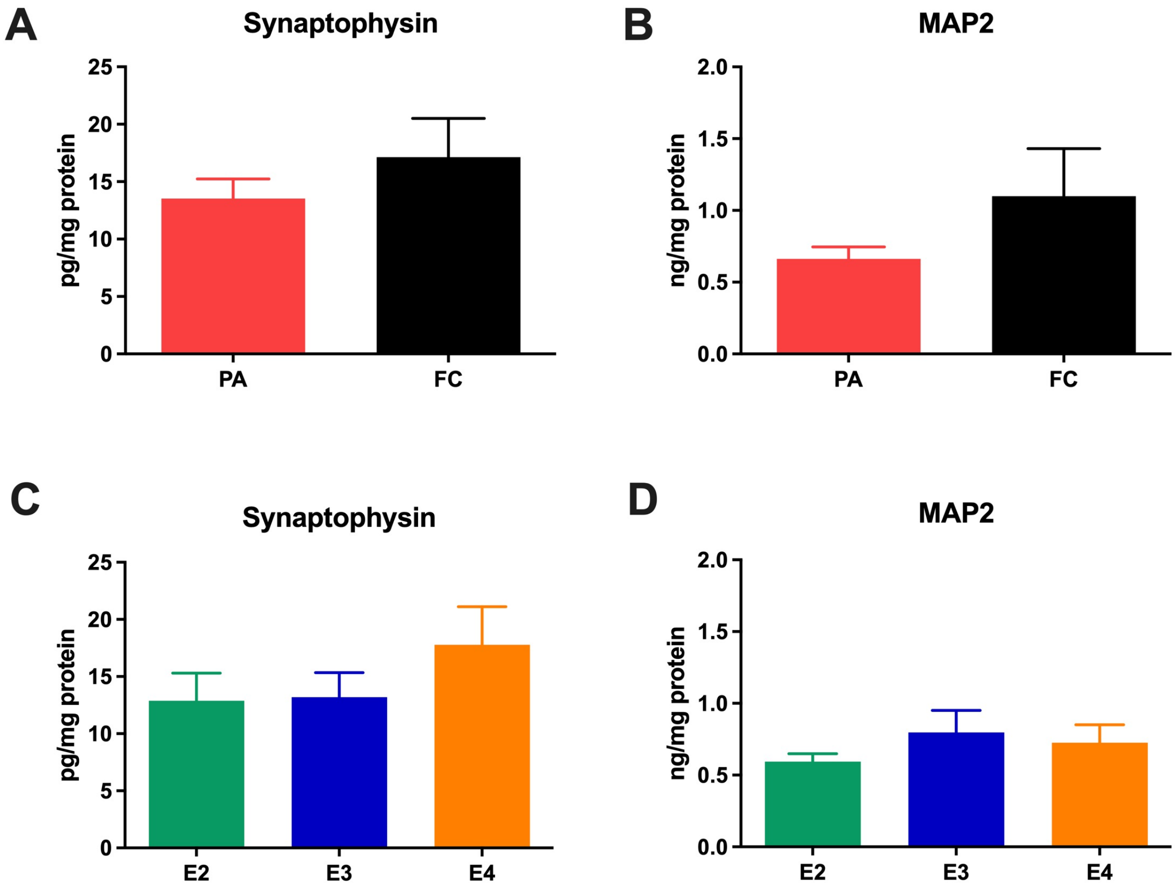

Fig. S7. A. Hippocampal Syn levels were comparable in mice tested for passive avoidance (PA) and fear conditioning (FC) (*n* = 21 mice (PA); *n* = 9 mice (FC)). **B.** Hippocampal MAP2 levels were comparable in mice tested for passive avoidance (PA) and fear conditioning (FC) (*n* = 21 mice (PA); *n* = 12 mice (FC)). **C.** Hippocampal Syn levels were comparable in E2, E3, and E4 mice tested for passive avoidance or fear conditioning (*n* = 11 mice (E2); *n* = 9 mice (E3), and *n* = 10 mice (E4)). **D.** Hippocampal MAP2 levels were comparable in E2, E3, and E4 mice tested for passive avoidance or fear conditioning (*n* = 11 mice (E2); *n* = 11 mice (E3), and *n* = 10 mice (E4)).

A
B
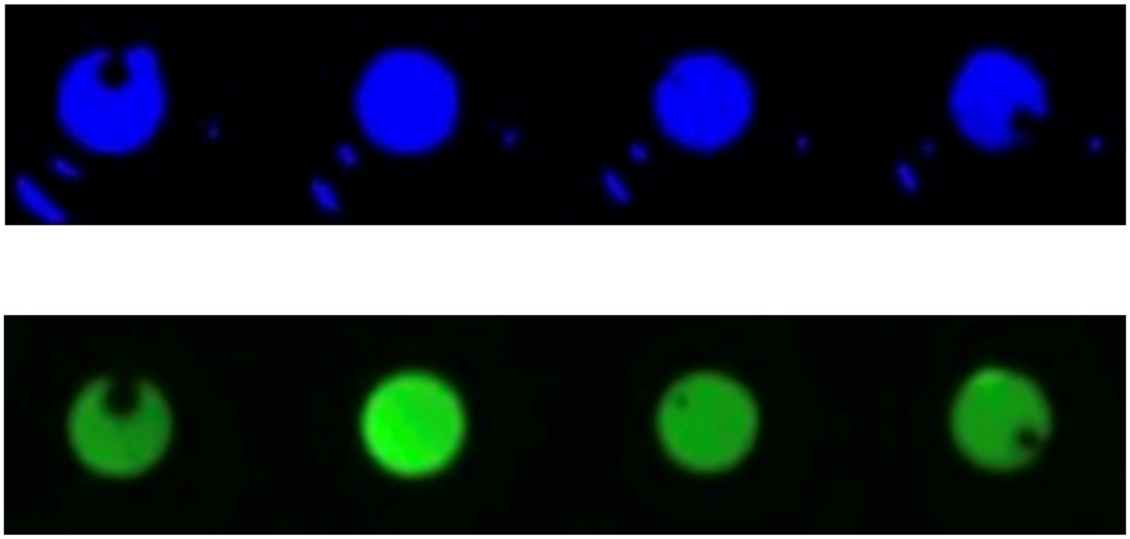

Fig. S8. Sample images of the tau (A) and total protein (B) images in the dot blot analysis.
